# Bleogens: Cactus-Derived Anti-Candida Cysteine-Rich Peptides with Three Different Precursor Arrangements

**DOI:** 10.3389/fpls.2017.02162

**Published:** 2017-12-22

**Authors:** Shining Loo, Antony Kam, Tianshu Xiao, James P. Tam

**Affiliations:** School of Biological Sciences, Nanyang Technological University, Singapore, Singapore

**Keywords:** bleogens, biosynthesis, cactus, cysteine-rich peptide, natural product, proteomics, peptides, plant

## Abstract

Cysteine-rich peptides (CRPs) play important host-defense roles in plants. However, information concerning CRPs in the Cactaceae (cactus) family is limited, with only a single cactus-derived CRP described to date. Here, we report the identification of 15 novel CRPs with three different precursor architectures, bleogens pB1-15 from *Pereskia bleo* of the Cactaceae family. By combining proteomic and transcriptomic methods, we showed that the prototype, bleogen pB1, contained 36 amino acid residues, a six-cysteine motif typical of the six-cysteine-hevein-like peptide (6C-HLP) family, and a type I two-domain precursor consisting of an endoplasmic reticulum (ER) and a mature domain. In contrast, the precursors of the other 14 bleogens contained a type II three-domain architecture with a propeptide domain inserted between the ER and the mature bleogen domain. Four of these 14 bleogens display a third type of architecture with a tandemly repeating bleogen domain. A search of the Onekp database revealed that <1% plant species possess three different precursor architectures for the biosynthesis of 6C-HLPs, including *Lophophora williamsii, Pereskia aculeate, Portulaca cryptopetala, Portulaca oleracea, Portulaca suffruticosa*, and *Talinum* sp. NMR analysis confirmed that bleogen pB1 has cystine-knot disulfide connectivity as well as a two-beta-sheet and a four-loop structural fold that is similar to other 6C-HLPs. Sequence analysis, structural studies, and *in silico* modeling revealed that bleogen pB1 has a cation-polar-cation motif, a signature heparin-binding motif that was confirmed by heparin affinity chromatography. Cell-based assays showed that bleogen pB1 is non-toxic to mammalian cells but functions as an anti-Candida peptide. Taken together, our findings provide insight into the occurrence, functions and precursor architectures of CRPs in the cactus family.

## Introduction

*Pereskia bleo* (Kunth) DC, also known as rose cactus, is an herb belonging to the Cactaceae family that is commonly used in Southeast Asia. *Pereskia bleo* is a small, thorny shrub native to the western United States and South America that is distributed in tropical and subtropical regions (Zareisedehizadeh et al., 2014). In southeast Asian countries such as Singapore and Malaysia, *Pereskia bleo* leaves are traditionally consumed to treat hypertension, diabetes, cancers and inflammatory diseases (Tan et al., 2005; Er et al., 2007; Malek et al., 2009; Wahab et al., 2009; Zareisedehizadeh et al., 2014; Guilhon et al., 2015), as well as gastritis, hemorrhoids, ulcers, and wounds (Pinto Nde and Scio, 2014; Zareisedehizadeh et al., 2014). Thus far, the reported putative bioactive compounds of *Pereskia bleo* are limited to small molecule metabolites, including sterols, flavonoids, and carotenoids (Zareisedehizadeh et al., 2014).

Small molecule metabolites and proteins represent a major source of drug leads in natural products. In contrast, multiple disulfide-constrained peptides derived from medicinal plants represent an underexplored area in drug discovery. These plant-derived peptides, particularly the highly disulfide-crosslinked cysteine-rich peptides (CRPs) that have three to five disulfide bonds and a molecular weight between 2 and 6 kDa, are hyperstable, and could provide a potential source of leads for drug development in the neglected chemical space between small molecule metabolites and proteins (Nguyen et al., 2013). Functionally, many CRPs are known to be plant defense molecules, which act as antimicrobials, insecticides (Broekaert et al., 1995, 1997; Silverstein et al., 2007; Odintsova and Egorov, 2012; Nawrot et al., 2014), proteinase inhibitors (Hamato et al., 1995; Nguyen et al., 2014, 2015a; Loo et al., 2016), or immune-stimulating agents (Nguyen et al., 2016).

Plant-derived CRPs are ribosomally synthesized peptides that are processed from precursors encoded in multigene families (Farrokhi et al., 2008; Tam et al., 2015; Tavormina et al., 2015). CRPs are classified into different families based on their cysteine content, cysteine spacing and disulfide connectivities (Tam et al., 2015). One of the most common cysteine motifs is the CX_n_CX_n_CCX_n_CX_n_C motif, which we have classified as the six-cysteine-hevein-like peptide (6C-HLP) family. The cysteine motif of hevein-like peptides was first discovered in hevein produced by the rubber tree (*Hevea brasiliensis*) that contains eight cysteines, cystine-knot disulfide connectivity, and a chitin-binding domain (Van Parijs et al., 1991). The 6C-HLPs are classified as hevein-like peptides because they share with heveins cystine-knot connectivity and the conserved chitin-binding domain. The 6C-HLP family has since expanded to include subfamilies that have a similar cysteine motif, but lack the chitin-binding domain. These subfamilies include cystine-knot antimicrobial peptides such as Mj-AMP1, Mj-AMP2, and PAFP-S (Cammue et al., 1992; Gao et al., 2001), cystine-knot alpha-amylase inhibitors such as allotide (Nguyen et al., 2015a), alstotides (Nguyen et al., 2015b) and wrightides (Nguyen et al., 2014), and neutrophil elastase inhibitors such as roseltides (Loo et al., 2016). The precursor architectures observed in the 6C-HLP family can be broadly classified into two types: type I biosynthetic precursors, found in the cystine-knot antimicrobial peptides that contain two domains, a signal and a mature peptide (Tam et al., 2015); and type II precursors, found in roseltides and cystine-knot alpha-amylase inhibitors, which contain three domains, with a pro-domain inserted between the signal peptide and a mature peptide domain (Nguyen et al., 2014, 2015a,b; Loo et al., 2016). A variation of the three-domain architecture (type IIa) is found in the chitin-binding 6C-HLPs such as altides, which contain a signal peptide, a mature peptide, and a C-terminal domain (Kini et al., 2015).

In a mass-spectrometry-driven profiling and discovery program to identify CRPs in medicinal plants, we found a cluster of CRPs ranging from 3 to 5 kDa from aqueous extracts of the medicinal cactus plant *Pereskia bleo*, belonging to the Cactaceae family. To date, there is only one cactus-derived CRP (Ep-AMP1), which is a 6C-HLP isolated from *Echinopsis pachanoi*, a medicinal plant found in South America (Aboye et al., 2015). Herein, we report the identification and characterization of a novel anti-Candida CRP, bleogen pB1, along with 14 other bleogens from *Pereskia bleo*, by both proteomic and transcriptomic methods. We showed that these bleogens belong to the 6C-HLP family but display three different types of biosynthetic precursors, one of which contains a tandemly repeating mature domain (type IIb). We also showed that bleogen pB1 is a novel heparin-binding anti-Candida CRP.

## Results

### Identification of Cysteine-Rich Peptides from *Pereskia bleo*

**FIGURE 1:**
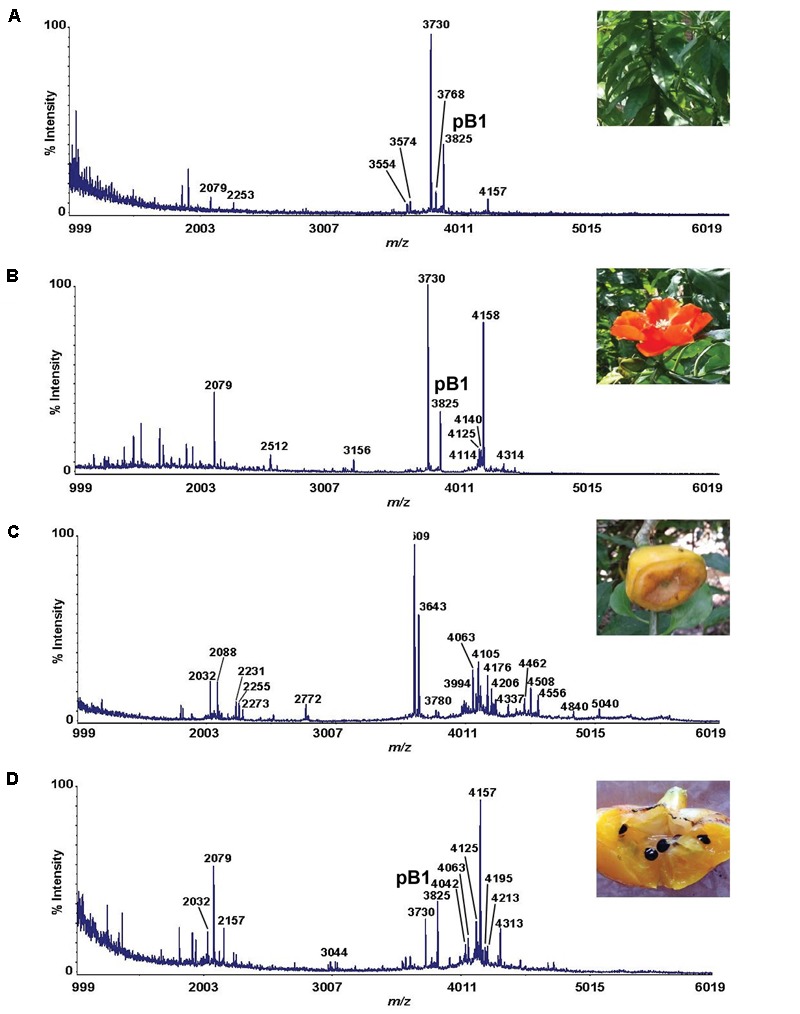
MS profiles of different parts of *Pereskia bleo* plants. **(A)** leaves, **(B)** flowers, **(C)** fruits, and **(D)** seeds from *Pereskia bleo* were collected and profiled using MALDI-TOF MS.

Mass spectrometry-driven profiling of aqueous extracts of *Pereskia bleo* leaves, flowers, fruits and seeds revealed the presence of a cluster of strong signals in the m/z range of 3000–5000 (**Figure 1**). The dominant m/z 3825 peak, designated as bleogen pB1, was isolated and subjected to *S-*reduction and *S-*alkylation using dithiothrietol (DTT) and iodoacetamide (IAM), respectively. After *S-*reduction and *S-*alkylation, bleogen pB1 displayed a m/z increase of 348, indicating that it is a CRP with six cysteine residues (Supplementary Figure S1).

### Primary Sequence and Biosynthesis of Bleogen pB1

Since bleogen pB1 is one of the major CRP in *Pereskia bleo* leaf, flower and seed extracts, it was selected for purification and characterization in a scaled-up aqueous extract of *Pereskia bleo* leaves. The crude aqueous extracts were first fractionated by C18 reversed-phase and strong cation-exchange flash chromatography, followed by ultrafiltration using a membrane with a 2000 Da molecular weight cut-off. The CRP-concentrated fraction was further purified by reversed-phase high performance liquid chromatography (RP-HPLC) (Supplementary Figure S2). From one kg of fresh leaves, 5–10 mg of purified bleogen pB1 was obtained.

**FIGURE 2:**
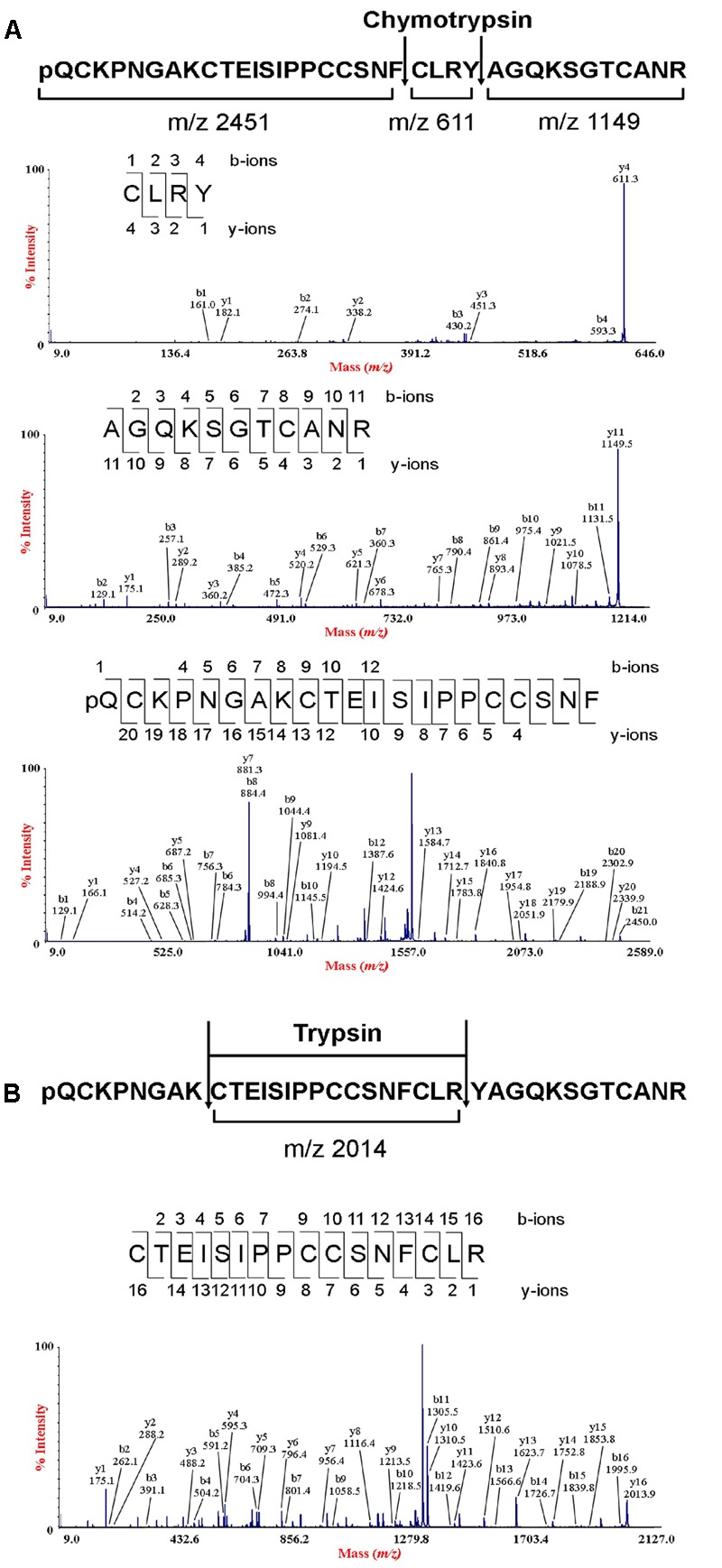
*De novo* sequencing of bleogen pB1. **(A)** *S*-alkylated bleogen pB1 was digested with chymotrypsin to yield three fragments with *m/z* 2451, 611 and 1149; and MS/MS spectra of m/z 2451, 611 and 1149 fragments; **(B)** *S*-alkylated bleogen pB1 was digested with trypsin at two sites, yielding fragments with *m/z* 2014 and MS/MS spectra of a m/z 2014 fragment.

**FIGURE 3:**
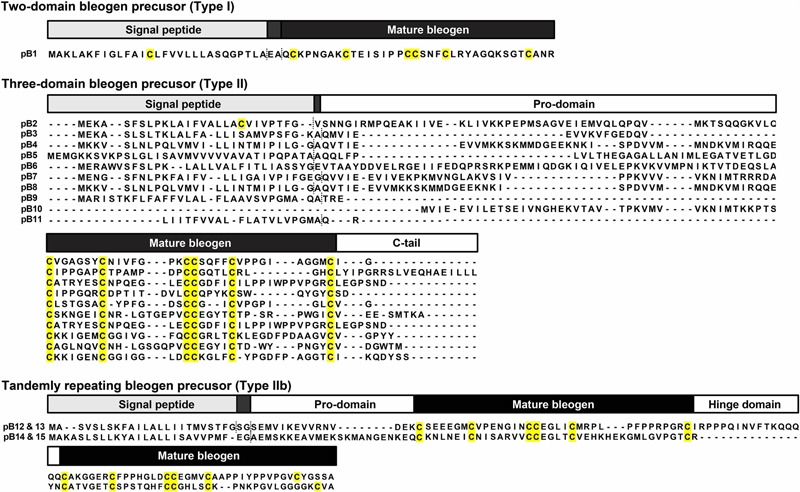
Three different precursor architectures for bleogens pB1-15. Dash lines represent signal peptide cleavage site as predicted by SignalP V4.1 and Phobius server. Type I: Two domain precursor (Signal peptide – mature peptide); Type II: Three domain precursor (Signal peptide – Pro-domain – Mature peptide); Type IIa: Three domain precursor (Signal peptide – Mature peptide – C-terminal tail/protein cargo); Type IIb: Tandemly repeating precursor (Signal peptide – Pro-domain – Mature peptide – Hinge domain – Mature peptide).

To determine the amino acid sequence of bleogen pB1, the purified bleogen pB1 was fully *S*-reduced and *S*-alkylated, and then digested with either trypsin or chymotrypsin. The resulting peptide fragments were analyzed by MALDI-TOF MS, followed by MS/MS sequencing. Analysis using the generated *b*-ions and *y*-ions revealed that bleogen pB1 is a 36-amino-acid-residue peptide with six cysteines (**Figure 2**). Transcriptomic analysis confirmed the amino acid sequence of bleogen pB1, which was biosynthesized as a 67-residue precursor with two-domains: a 29/31-residue N-terminal signal peptide and a 38/36-residue C-terminal mature peptide as predicted by SignalP V4.1 (Petersen et al., 2011) and the Phobius server (Kall et al., 2007), respectively (**Figure 3**). Transcriptomic analysis further identified 14 other bleogens (pB2 to pB15) having different precursor architectures that included a three-domain precursor and a tandemly repeating bleogen domain (**Figure 3**).

### NMR Structure of Bleogen pB1

To characterize the structural fold of bleogen pB1, its solution structure was determined from 2D ^1^H, ^1^H- TOCSY and NOESY NMR spectra. The sequential assignment was performed based on the NOE cross peaks between HN_i_ and Hα_i-1_ as well as the other side chain protons of residue i-1 (Supplementary Figures S3, S4). Amide protons of residue i have NOE cross peaks with the side chain protons of the residue i-1. The pattern of the peaks in TOCSY of each amide proton stripe provided information on the amino acid type. Based on these strategies, sequential amino acid assignment was completed. More than 95% of the peaks in the NOESY spectrum could be assigned unambiguously.

To confirm disulfide bond connectivity, lowest-energy analysis was performed to compare the averaged energies of the structures generated with the different disulfide bond patterns assumed for structure calculation using CNSsolve 1.3 (Brunger et al., 1998). The lowest number of NOE violations of the correct disulfide connectivity will have the lowest averaged energy. Pattern 1 (Supplementary Figure S4) exhibited the lowest averaged energy, suggesting that the disulfide bond connectivities are Cys2–Cys18, Cys9–Cys22, and Cys17–Cys33 (Pattern 1). For Pattern 1, the 20 best structures among 100 structures were highly converged, with backbone RMSD and heavy atom RMSD of 0.67 ± 0.32 Å and 1.30 ± 0.36 Å, respectively (**Table 1**). Further evidence supporting the Cys2–Cys18, Cys9–Cys22, and Cys17–Cys33 disulfide linkages was obtained from the Hβ-Hβ NOE cross peaks (Supplementary Figure S5).

**Table 1:** Restraints and structure statistics of bleogen pB1.

Experimental restraints and structural statistics of 20 lowest-energy structures of bleogen pB1 among the 100 structures generated by CNSsolve 1.3		
NMR distance restraints	396	
Intra-residue NOE (|*i*-*j*| = 0)	107	
Sequential NOE (|*i*-*j*| = 1)	115	
Medium-range NOE (1 < |*i*-*j*|≤ 5)	47	
Long-range NOE (|*i*-*j*| > 5)	122	
Hydrogen bond	5	
Diherdral angle restraints	6	
Structural statistics (36 residues,Q^1^-R^36^)		
Violations per structure		
NOE violation (Å)		0.025 ± 0.002
Maximum NOE violation (Å)		0.030
Dihedral angle violation (°)		0.225 ± 0.066
Maximum NOE violation (°)		0.329
Ramachandran plot region (36 residues)		
Residues in most favored regions	18	64.3%
Residues in additional allowed regions	8	28.6%
Residues in generously allowed regions	2	7.1%
Residues in disallowed regions	0	0%
Number of end-residues (excl. Gly and Pro)	2	
Number of glycine residues	3	
Number of proline residues	3	
Mean RMSD from the average coordinates (36 residues, Q^1^-R^36^)		
Backbone atoms(Å)		0.67 ± 0.32
Heavy atoms(Å)		1.30 ± 0.36

**FIGURE 4:**
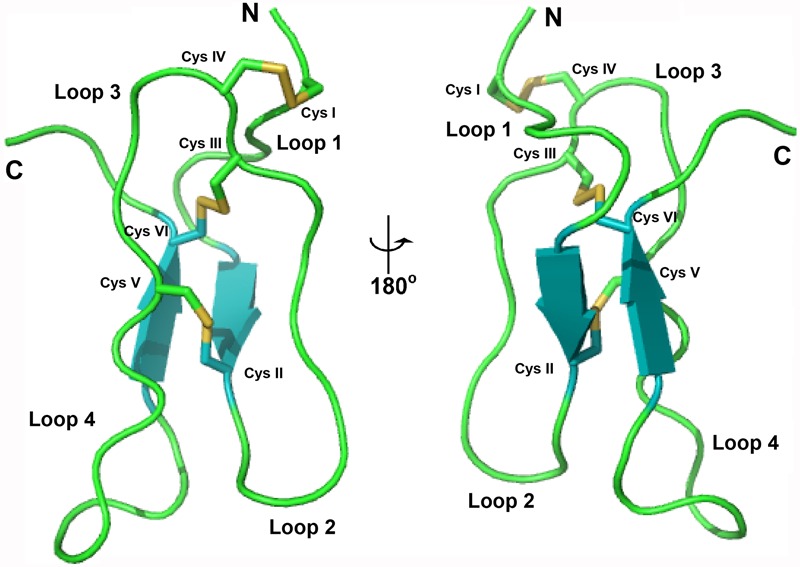
Solution structure of bleogen pB1.

The structure of bleogen pB1 (PDB entry: 5xbd), generated by simulated annealing, contains one loop and two antiparallel β strands, ranging from Ala7-Cys9 and Gly31-Cys33. The three prolines adopt a *trans* form. This structure was supported by NOE cross peaks between Hδ_i_ and HN_i-1_ of the proline residue and the previous residue, respectively. Bleogen pB1 contains two adjacent cysteines, Cys17 and Cys18, which have side chains that are oriented in opposite directions. The disulfide bond Cys2–Cys18 causes the N terminus of the peptide to be anchored to the loop, whereas the Cys9–Cys22 and Cys17–Cys33 disulfide bonds intersect in the center of bleogen pB1 (**Figure 4**). Overall, bleogen pB1 exists as a four-looped CRP stabilized by three disulfide bonds and two antiparallel β strands, a structural fold shared by many 6C-HLPs (Broekaert et al., 1995; Nguyen et al., 2014, 2015a,b; Tam et al., 2015).

### Bleogen pB1 Exhibits Heparin Binding Activity

**FIGURE 5:**
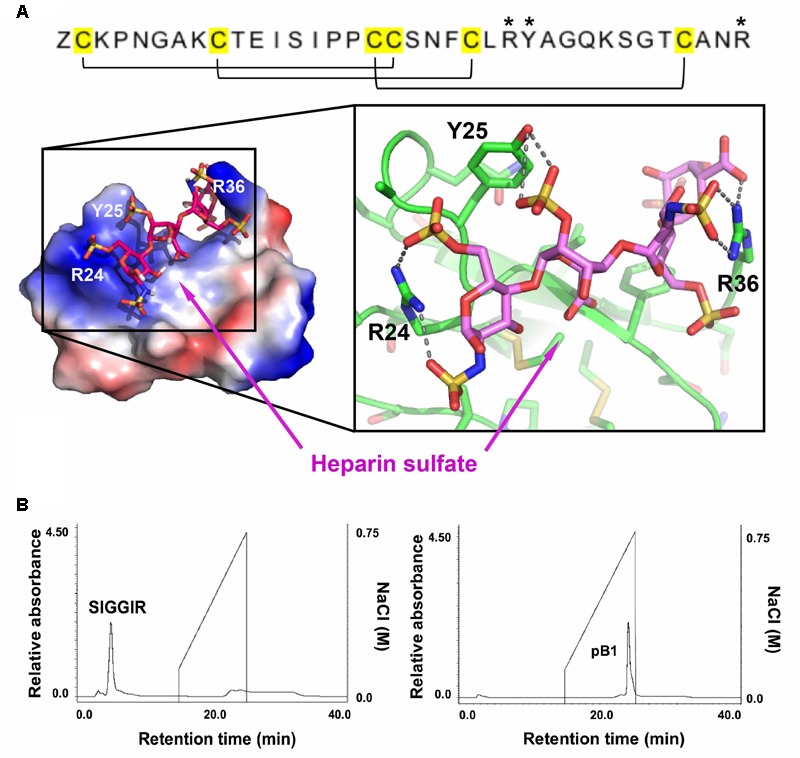
Heparin-binding properties of bleogen pB1. **(A)** Modeling the interaction between bleogen pB1 and heparin sulfate using the ClusPro Version 2.0 server. Blue: electropositively charged, Red: electronegatively charged and White: Neutral. **(B)** Bleogen pB1 was retained on a heparin HPLC affinity column and required around 0.3 M NaCl for elution. A model peptide (SIGGIR) was not retained on the column. ^∗^Denote the amino acids essential for the heparin-binding motif.

Sequence analysis and the NMR structure of bleogen pB1 revealed a positively charged clamp formed by two arginine residues (R24 and R36) and a tyrosine residue (Y25). The average measured distances between the α-carbons were 14.2 Å (R24–R36), 15.2 Å (R24–Y25), and 3.8 Å (Y25–R36) (Supplementary Figure S6). The spatial arrangement of these three residues forms a distinctive interacting surface known as the cation-polar-cation (CpC) clip motif, a structural signature of heparin-binding proteins (Torrent et al., 2012). *In silico* docking of heparin was performed using the automatic protein-protein docking server ClusPro Version 2.0 operating in heparin mode (Mottarella et al., 2014). The negatively charged heparin sulfate interacts with the two basic residues (R24 and R36) and polar residue (Y25) in bleogen pB1 (**Figure 5A**). The heparin-binding activity of bleogen pB1 was confirmed by heparin affinity chromatography showing that bleogen pB1 binds to a heparin HPLC affinity column and can be eluted with 0.3 M NaCl (**Figure 5B**). In contrast, a model peptide (SIGGIR) did not bind to the heparin affinity column. Using heparin-affinity chromatography, we could improve the extraction yield of bleogen pB1 by fourfold, to obtain 20–40 mg of bleogen pB1 per kg of fresh leaves.

### Bleogen pB1 Is an Anti-Candida Peptide

**FIGURE 6:**
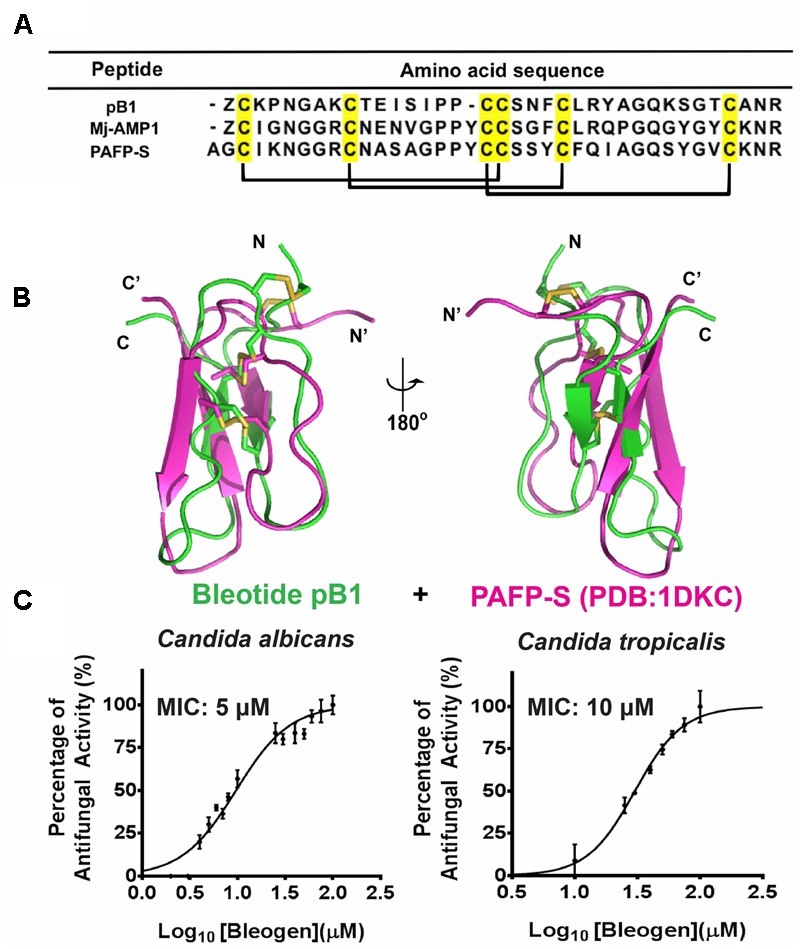
**(A)** Sequence alignment of Knottin-type anti-microbial peptide with bleogen pB1; MJ-AMP1 from *Mirabilis jalapa*; PAFP-S and Pa-ASP from *Phytolacca americana*; **(B)** Superposition of bleogen pB1 (green) on PAFP-S (PDB entry: 1DKC; purple). **(C)** Anti-microbial activities of bleogen pB1 against *Candida albicans* and *Candida tropicalis*. Minimal inhibition concentration (MIC) was determined using a radial diffusion assay. All results were expressed as mean ± S.E.M (*n* = 3). Amphotericin B was used as a positive control.

To predict the possible functions of bleogen pB1, a pBLAST search of the NCBI database was conducted. The search results showed that bleogen pB1 shares sequence similarity with antimicrobial peptides such as MJ-AMP1 from *Mirabilis jalapa* and PAFP-S from *Phytolacca americana* (Cammue et al., 1992; Gao et al., 2001; **Figure 6A**). Protein tertiary structure comparison with PAFP-S (PDB entry: 1DKC) conducted using SuperPose software Version 1.0 (Maiti et al., 2004) showed that PAFP-S has a similar structural fold as bleogen pB1 (**Figure 6B**). The RMSD values between the superimposed structures of PAFP-S and bleogen pB1 were 0.681 Å and 1.292 Å for all Cα and heavy atoms, respectively, suggesting that bleogen pB1 could possess antimicrobial activities.

A radial diffusion assay was conducted to evaluate the antimicrobial activities of bleogen pB1 using several bacterial strains (*Escherichia coli, Staphylococcus epidermidis, Staphylococcus aureus* and *Enterococcus faecium*) and fungal strains (*Candida albicans* and *Candida tropicalis*). Bleogen pB1 displayed antifungal activities toward *Candida albicans* and *Candida tropicalis* with minimal inhibitory concentration (MIC) of 5 and 10 μM, respectively (**Figure 6C**). No antibacterial activity was observed in the tested bacterial strains. Since high-salt solutions (>0.3 M NaCl) can elute bleogen pB1 from heparin affinity columns, we next examined the anti-Candida activities of bleogen pB1 in a radial diffusion assay under high-salt conditions. Bleogen pB1 did not display anti-Candida properties in the presence of 0.3 M NaCl. Moreover, propidium iodide staining showed that bleogen pB1 is not membrane lytic (Supplementary Figure S7).

### Bleogen pB1 Is Not Cytotoxic or Hemolytic

**FIGURE 7:**
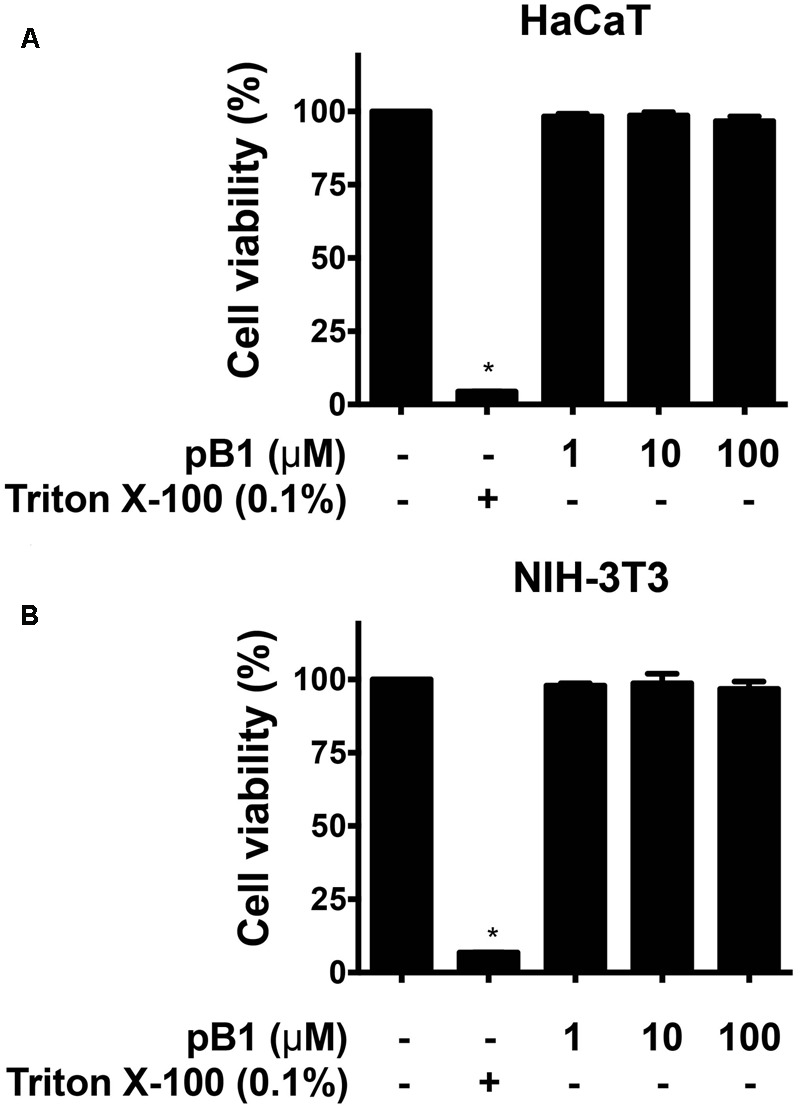
Bleogen pB1 does not show cytotoxic activities. Effects of bleogen pB1 on **(A)** HaCaT and **(B)** NIH-3T3 cells. All results were expressed as mean ± S.E.M. (*n* = 3). ^∗^*P* < 0.05 compared to control group.

To determine the cytotoxicity of bleogen pB1, cell viability was measured by MTT assay. Treatment of HaCaT (human keratinocyte cells) or NIH-3T3 cells (mouse fibroblast cells) with bleogen pB1 at concentrations up to 100 μM for 24 h did not affect cell viability (**Figure 7**). Bleogen pB1 at up to 100 μM also did not show hemolytic effects.

### Onekp Database Search for Bleogen Homologous Genes with Two Domain Precursors

**FIGURE 8:**
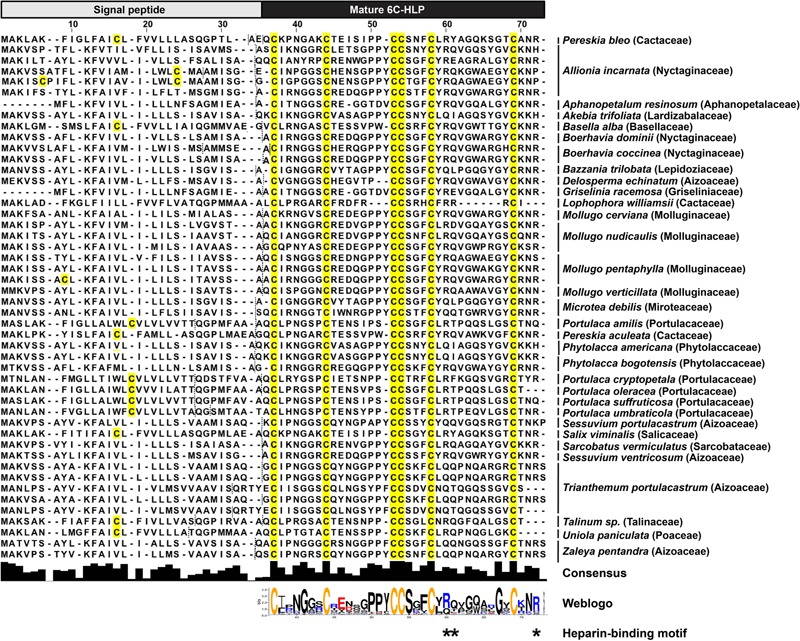
Alignment of 47 two-domain precursor sequences with three disulfide bonds and a cysteine motif of CX_n_CX_n_CCX_n_CX_n_C by tblastn search of the Onekp database. Dash lines represent signal peptide cleavage site as predicted by SignalP V4.1 and Phobius server.

To explore the diversity of the two-domain precursor architecture of bleogen pB1, we performed a tblastn search in Onekp, a comprehensive plant transcriptome database of 1000 plants, using the precursor sequence of bleogen pB1. Based on our database search, we identified 47 other two-domain precursor sequences with three disulfide bonds and a cysteine motif of CX_n_CX_n_CCX_n_CX_n_C from 32 plant species in 17 families (**Figure 8**). Of the 32 plant species, six plants from three different families contained both three-domain and tandemly repeating precursor sequences found in the Onekp database. These plants included *Pereskia aculeata* and *Lophophora williamsii* from the Cactaceae family; *Portulaca cryptopetala, Portulaca oleracea*, and *Portulaca umbraticola* from the Portulacaceae family; and *Talinum sp* from the Talinaceae family (Supplementary Figures S8, S9).

## Discussion

This report identified novel cactus-derived CRPs, bleogen pB1 and 14 other bleogens, from the medicinal plant *Pereskia bleo.* Our work greatly expands the family of cactus-derived CRPs, as Ep-AMP1 from *Echinopsis pachanoi* was the only previously known example. A comparison of Ep-AMP1 and bleogen pB1 primary sequences showed that, apart from the six conserved cysteine residues arranged in the cysteine motif CX_n_CX_n_CCX_n_CX_n_C, the remaining 25 amino acids in the sequence were not conserved.

The 6C-HLP family of CRPs carries the evolutionarily conserved CX_n_CX_n_CCX_n_CX_n_C cysteine motif (Nguyen et al., 2014, 2015a,b; Aboye et al., 2015; Kini et al., 2015; Tam et al., 2015; Loo et al., 2016) and can be further divided into two subfamilies based on the presence or absence of a chitin-binding domain. Six-cysteine-containing CRPs having a tandemly connecting CC motif at the Cys III and Cys IV positions are generally arranged as a cystine-knot with disulfide connectivity of Cys I–IV, Cys II–V, and Cys III–VI (Nguyen et al., 2014, 2015a,b; Aboye et al., 2015; Tam et al., 2015; Loo et al., 2016). In the 6C-HLP family, the four-looped scaffold is highly conserved, whereas the amino acid residues in the inter-cysteine loops are highly variable (Tam et al., 2015; Loo et al., 2016). The absence of a chitin-binding domain in bleogens classifies them in the non-chitin-binding subfamily of 6C-HLPs.

The 6C-HLPs have two general types of precursor architectures. Type I has a two-domain precursor comprised of a signal peptide and a mature 6C-HLP domain that is seen in Mj-AMP-1, Mj-AMP2 and PAFP-S precursors (Cammue et al., 1992; Gao et al., 2001). Meanwhile, type II has a three-domain precursor comprised of a signal peptide, a pro-domain, and the mature 6C-HLP domain with or without a short C-terminal tail (as shown in roseltide and CKAI precursors) (Nguyen et al., 2014, 2015a,b; Loo et al., 2016). A variation of the three-domain architecture (type IIa) is seen in chitin-binding 6C-HLPs such as altides (Kini et al., 2015) that contain a signal peptide, a mature peptide, and a C-terminal domain, which can be a protein or a short C-terminal tail. Transcriptome analysis revealed that the 6C-HLP bleogen pB1 has a type I two-domain precursor comprising an ER signal peptide and a mature domain. Interestingly, 14 other bleogens (pB2–pB15) having a similar cysteine spacing pattern were found to be derived from two different types of precursors. The pB2-pB11 precursors contain a type II three-domain arrangement, whereas pB12–pB15 precursors contain a variation of a type II three-domain arrangement with a tandemly repeating bleogen domain (type IIb). A search of the Onekp database that includes 1000 plant transcriptomes revealed that less than 1% of the plant species contain three different precursor architectures for 6C-HLP biosynthesis (**Figure 8** and Supplementary Figures S8, S9). Thus, *Pereskia bleo* is an unusual plant that synthesizes three different 6C-HLP precursor architectures.

In plants, the type I two-domain precursor architectures can be observed in several CRP families, including plant defensins, lipid transfer proteins, and 6C-HLPs (Cammue et al., 1992; Broekaert et al., 1995, 1997; Gao et al., 2001; Tam et al., 2015) that largely function as antimicrobial peptides. For 6C-HLPs, Mj-AMP1, and Mj-AMP2 from *Mirabilis jalapa* (Cammue et al., 1992), and PAFP-S from *Phytolacca americana* (Gao et al., 2001) are three examples that were reported to have antimicrobial activities and are bioprocessed from a two-domain precursor. To gain insights into the distribution and diversity of 6C-HLPs with two-domain precursor sequences from plants, a search of the OneKP database was performed using the bleogen pB1 precursor sequence. This search yielded 47 precursor sequences derived from 17 different plant families of gymnosperms and angiosperms (**Figure 8**). Of the 47 precursor sequences, the mature peptides of 45 precursor sequences were observed to be rich in Lys and Arg residues. This characteristic is similar to the mature peptides of Mj-AMP1, Mj-AMP2, PAFP-S, and bleogen pB1, which all possess antimicrobial activities. Hence, we speculated that the type I two-domain precursor architecture could be a common feature for cationic 6C-HLPs that have antimicrobial activities.

An additional feature of antimicrobial 6C-HLPs appears to be the CpC clip motif, which is characterized by a conserved pattern comprising one polar and two positively charged residues. The spatial arrangement of these residues defines the distances between the cationic and polar residues (Torrent et al., 2012). In bleogen pB1 the amino acids R24, Y25, and R36 form the CpC clip motif. *In silico* modeling showed that the bleogen pB1 heparin-binding pocket also includes R24, R36, and Y25 that interact with negatively charged heparin sulfate through ionic and hydrogen bonds. Torrent et al. (2012) proposed that the CpC clip motif is a structural signature of heparin-binding proteins, including basic fibroblast growth factor, thrombin, RANTES, CCL5, and the cobra cardiotoxin A3. This motif is believed to be the primary attachment site of heparin or other sulfated glycosaminoglycans to heparin-binding proteins. Heparan sulfate proteoglycans (HSPG) are glycoproteins characterized by a protein core with multiple covalently attached heparan sulfate chains (Tumova et al., 2000). Heparan sulfate belongs to the glycosaminoglycan family of sulfated polysaccharides, whereas heparin sulfate is the highly sulfated form of heparan sulfate (Tumova et al., 2000). Using heparin affinity chromatography, we showed that bleogen pB1 has heparin-binding properties. This heparin-binding property also facilitated bleogen pB1 isolation and purification to enhance the yield from 10 to 40 mg per kg of fresh leaves. Our Onekp database search identified 29 out of the 47 two-domain precursor sequences, together with Mj-AMP1, Mj-AMP2, PAFP-S that possess the CpC clip motif in the last inter-cysteine loop (loop 4) and a C-terminal region that has heparin-binding properties (**Figure 8**). Thus, we envision that heparin-affinity chromatography can be used to identify and purify potentially bioactive heparin-binding CRPs from plant extracts.

Bleogen pB1 exerts selective antimicrobial activities against the two tested Candida strains with MICs in the micromolar range. However, the finding that high-salt conditions inhibited bleogen pB1 anti-Candida activity suggests that ionic interactions are likely important and that the heparin-binding property of bleogen pB1 is essential for its anti-Candida properties. Indeed, a previous report showed that endogenous heparin-binding peptides, including LL-37 and alpha-defensins, exerted antimicrobial activities against *Candida albicans* (Schmidtchen et al., 2001, 2002). Bleogen pB1 is not membranolytic to human cells as shown in a hemolytic assay and propidium iodide staining (Supplementary Figure S7). Thus, the anti-Candida activity of bleogen pB1 likely does not occur through a pore-formation mechanism. Further studies are required to understand the intracellular target(s) responsible for the anti-Candida properties of bleogen pB1.

## Conclusion

Here, we showed that *Pereskia bleo* of the cactus family produces 6C-HLPs with three different types of precursor architectures. This study greatly expands our knowledge of the occurrence, functions and precursor architectures of 6C-HLPs. Our results also highlight the discovery of bleogen pB1 as the first plant-derived heparin-binding anti-Candida 6C-HLP from the Cactaceae family.

## Experimental Procedures

### Materials

All chemicals and solvents, unless otherwise stated, were purchased from Sigma–Aldrich (St. Louis, MO, United States) and Fisher Scientific (Waltham, MA, United States).

### Plant Materials

Samples of fresh *Pereskia bleo* leaves, flowers, fruits and seeds were collected from the Nanyang Community Herb Garden, Nanyang Technological University, Singapore (courtesy of Mr. Ng Kim Chuan). Sample authenticity was determined taxonomically by S. Lee and H.J. Lam of the Singapore Botanic Gardens and voucher specimens were deposited at the Singapore Herbarium in Singapore Botanic Gardens (code number: SING 2015-145).

### Screening and Profiling

Fresh *Pereskia bleo* plant parts were extracted with water for 15 min at room temperature at a 1:10 ratio. The aqueous extracts were vortexed vigorously and centrifuged at 16000 × g for 5 min at 4°C before being subjected to flash chromatography using C18 solid phase extraction (SPE) columns (Waters, United States). The fractions were eluted with 60% ethanol and analyzed by matrix-assisted laser desorption/ionization-time of flight mass spectrometry (MALDI-TOF MS; AB SCIEX 4700 MALDI-TOF/TOF). The MALDI spectra were acquired in the m/z range of 500–6000, with a focus m/z 3500. Total laser shots were 2250 with a laser intensity of 3500.

### Heparin Affinity Chromatography

Heparin binding chromatography was performed by high performance liquid chromatography (HPLC; Shimadzu, Japan). A linear gradient of mobile phase A (10 mM phosphate buffer, pH 7.2) and mobile phase B (0.75 M NaCl in 10 mM phosphate buffer, pH 7.2) was used with a TSKgel Heparin-5PW column (75 mm × 75 mm, 10 μm; Tosoh Bioscience, Japan).

### *S*-Reduction and *S*-Alkylation

Purified bleogen pB1 was *S*-reduced by 20 mM dithiothreitol (DTT) in ammonium bicarbonate buffer (25 mM) pH 8 at 37°C for 30 min, followed by *S*-alkylation with 200 mM IAM at 37°C for 60 min. MALDI-TOF MS was used to confirm the mass shift after *S*-reduction and *S*-alkylation. The MALDI spectra were acquired in the m/z range of 500–6000, with a focus m/z 3500. Total laser shots were 2250 with a laser intensity of 3500.

### Scale-up Extraction and Purification of Bleogen pB1

Fresh *Pereskia bleo* leaves (1 kg) were blended for 15 min with water and centrifuged at 9000 rpm for 10 min at 4°C (Beckman Coulter, United States). The supernatant was filtered through 1 μM pore size glass fiber filter paper (Sartorius, Singapore). The filtrate was loaded onto a C18 flash column (Grace Davison, United States) and eluted with 60% ethanol. The eluted fractions were then loaded onto an SP Sepharose resin column (GE Healthcare, United Kingdom), eluted with 1 M NaCl (pH 3.0), followed by ultrafiltration (ViVaflow 200, 2000 MWCO hydrostat). Further purification was performed by RP-HPLC (Shimadzu, Japan). A linear gradient of mobile phase A (0.05% TFA/H_2_O) and mobile phase B (0.05% TFA/ACN) was used with a C18 column (250 mm × 22 mm, 5 μm, 300Å; Grace Davison, United States). MALDI-TOF MS was used to identify the presence of bleogen pB1 in the eluted fractions. The eluted fractions containing bleogen pB1 were lyophilized for storage at room temperature. The MALDI spectra were acquired in the m/z range of 500–6000, with a focus m/z 3500. Total laser shots were 2250 with a laser intensity of 3500.

### *De Novo* Peptide Sequencing

Purified *S*-alkylated bleogen pB1 (1 mg/mL) was digested with trypsin or chymotrypsin at a 5:1 (v/v) ratio in ammonium bicarbonate buffer (25 mM), pH 8 at 37°C for 10 min. The tryptic and chymotryptic peptide fragments were then analyzed by MALDI-TOF MS followed by MS/MS (AB SCIEX 4700 MALDI-TOF/TOF). *De novo* peptide sequencing was performed using the *b*-ions and *y*-ions. The MALDI spectra were acquired in the m/z range of 500–6000, with a focus m/z 3500. Total laser shots were 2250 with a laser intensity of 3500. The MALDI MS/MS spectra for the digested fragments of m/z 611, 1149 and 2451 for chymotryptic peptides, and m/z 2014 for tryptic peptides were acquired using a laser intensity of 5000 with a total of 8000 laser shots.

### Total RNA Isolation and Next Generation Transcriptome Sequencing

RNA isolation from fresh *Pereskia bleo* leaves was performed based on the protocol of Djami-Tchatchou and Straker (2011) using CTAB extraction buffer (2% cetyltrimethylammonium bromide, 2% polyvinylpyrrolidone, 100 mM Tris–HCl (pH 8.0), 2 mM EDTA, 2 M NaCl, 2% 2-mercaptoethanol; Djami-Tchatchou and Straker, 2011). RNA library construction was performed using 1 μg total RNA (RIN value > 7.0) with an Illumina TruSeq mRNA Sample Prep kit (Illumina Inc., United States). Briefly, poly-A containing mRNA molecules were purified using poly-T-attached magnetic beads. Following purification, mRNA fragmentation was performed using divalent cations at an elevated temperature. RNA fragments were reverse-transcribed into first strand cDNA using SuperScript II reverse transcriptase (Invitrogen) and random primers, followed by second strand cDNA synthesis using DNA Polymerase I and RNase H. These cDNA fragments were subjected to end repair processing, the addition of a single ‘A’ base, and ligation of the indexing adapters. The products were then purified and enriched using PCR to create the final cDNA library. The libraries were quantified using qPCR according to the qPCR Quantification Protocol Guide (KAPA Library Quantification kits for Illumina Sequencing platforms) and qualified using TapeStation D1000 ScreenTape (Agilent Technologies, Germany). Indexed libraries were sequenced using the HiSeq2500 platform (Illumina Inc., United States) and the reads were assembled using Trinity by Macrogen Inc. (Korea). The transcriptomic raw data were deposited at NCBI database under BioProject: PRJNA416167, BioSample: SAMN07943319.

### tBlastn Search of Bleogens from *Pereskia bleo* Leaves Transcriptome

tBlastn was used to search for 6C-HLPs with a cysteine spacing pattern of CXCXCCXCXC from *Pereskia bleo* leaves transcriptome using bleogen pB1 as a query sequence with expect value threshold 100 (BioEdit v7.2.6.1).

### Onekp Database Search for Bleogen-Like Precursor Sequences

tBlastn was used to search for bleogen-like precursor sequences from OneKp using bleogens as query sequences with expect value threshold 100.

### Signal Peptide Prediction

Signal peptide cleavage sites were predicted using SignalP 4.1 server (Default D-cutoff values) and Phobius server.

### NMR Spectroscopy and Structure Determination of Bleogen pB1

A sample of bleogen pB1 for NMR spectroscopy was prepared by dissolving the lyophilized peptide in PBS to yield a final peptide concentration of 3 mM. All NMR spectra were collected at a sample temperature of 298 K on a Bruker AVANCE II 600 MHz NMR spectrometer equipped with four RF channels and a 5 mm z-gradient TCI cryoprobe. Phase-sensitive two-dimensional ^1^H, ^1^H-TOCSY and NOESY spectra were recorded with a spectral width of 12 ppm. For water suppression, excitation sculpting with gradients was applied to all NMR experiments. TOCSY and NOESY spectra were obtained with mixing times of 80 and 200 ms, respectively. The proton chemical shifts were referenced to external sodium 2,2-dimethyl-2-silapentane-5-sulfonate (DSS). All measurements were recorded with 2,048 complex data points and zero-filled to 2048 × 512 data matrices. Time domain data in both dimensions were multiplied by a 90°-shifted squared sine bell window function prior to Fourier transformation. Baseline correction was applied with a fifth order polynomial. NMR data were acquired and processed by TopSpin (Bruker BioSpin). The NMR spectra were processed with NMRpipe (Delaglio et al., 1995). Sequence-specific assignments were achieved with 2D TOCSY and NOESY and NOEs were performed using SPARKY (Goddard and Kneller, 2004). Distance restraints were derived based on the intensities of NOE cross peaks, which were divided into three classes: strong, 1 < *d* ≤ 1.8; medium, 1.8 < *d* ≤ 3.4; weak, 3.4 < *d* ≤ 5. Three-dimensional structures were reconstructed using CNSsolve 1.3 (Brunger et al., 1998). The six cysteines were assumed to form disulfide bonds in the structure calculation. Structures were displayed with Chimera (Huang et al., 1996) and Pymol (Delano, 2002) and validated with the online server PDBsum (Laskowski et al., 2005). Accession code(s): PDB ID 5XBD, BMRB ID 36066.

### *In Silico* Modeling

The *in silico* docking of heparin binding was performed using the automatic protein-protein docking server ClusPro Version 2.0 (Comeau et al., 2004a,b) that includes an advanced option for heparin docking. The docking involves global rigid docking using a fast Fourier transform correlation approach. Two sets of 900 lowest energy structures (using electrostatic energy, van der Waals attractions and van der Waals repulsions) were retained. The second step involved clustering the retained structures using pairwise RMSD. The ten largest clusters were then refined by minimizing the energy of the complexes. Clusters ranked the highest displayed the most contacts with the protein.

### Bacterial and Fungal Strains

The bacteria and fungi used were: *Escherichia coli* ATCC^®^ 25922^TM^, *Staphylococcus epidermidis* ATCC^®^ 12228^TM^, *Staphylococcus aureus* ATCC^®^ 29213^TM^, *Enterococcus faecium* (courtesy of Professor Kimberly Kline, NTU), *Candida albicans* ATCC^®^ 900028^TM^, and *Candida tropicalis* (identified by Charles River Laboratories International, Inc., Singapore). All bacterial and fungal strains were cultivated in tryptic soy broth (TSB) and tryptic soy agar, with the exception of *Candida albicans*, which was cultivated in super optimal broth with catabolite repressor (SOC) and SOC agar. All cultured microbial strains were incubated at 37°C with shaking at 350 rpm.

### Radial Diffusion Assay (RDA)

Bleogen pB1 was screened for antimicrobial activity on bacterial (*Escherichia coli, Staphylococcus epidermidis, Staphylococcus aureus, Enterococcus faecium*) and fungal (*Candida albicans, Candida tropicalis*) strains using the protocol described by Steinberg et al. (Steinberg and Lehrer, 1997). Briefly, the microbial strains were subcultured in fresh broth, and allowed to reach log phase by incubating for 3 h at 37°C with shaking at 350 rpm. The strains were centrifuged at 1000 ×*g*, 4°C for 10 min and washed once with sterile 10 mM sodium phosphate buffer, pH 7.4. OD_620_ was adjusted to 0.1 and an optimal volume of culture was inoculated into the underlay agar (20X dilution of TSB or SOC with 1% agarose) and plated. Wells (1 mm × 1 mm) were punched into the underlay agars and bleogen pB1 (0.1, 1, 10, 25, 30, 40, 50, 60, 75, and 100 μM) was added to the wells. Triton X-100 (1%) was used as the positive control for bacterial strains and amphotericin B as the positive control for fungal strains. Samples were allowed to diffuse into the agar by incubating at 37°C for 3 h. Nutrient-rich overlay agar was made to coat the underlay agar. For high salt conditions, 0.3 M NaCl in nutrient rich overlay agar was prepared. The plates were incubated at 37°C overnight before the inhibition zone was measured and normalized to determine the MIC. All the experiments were repeated for three times.

### Cell Culture

HaCaT (human keratinocyte) and NIH-3T3 (mouse fibroblast) cells were cultured in Dulbecco’s modification of Eagle’s medium (DMEM) supplemented with 10% fetal bovine serum and 100 U/mL penicillin and streptomycin and grown in a 5% CO_2_ humidified incubator at 37°C.

### Cell Viability Assay

Cell viability was measured using a 3-(4,5-dimethyl-2-thiazolyl)-2,5-diphenyltetrazolium bromide (MTT) dye reduction assay. Briefly, cells were treated with 100 μM bleogen pB1 or 0.1% Triton X-100 (positive control) for 24 h. MTT (0.5 mg/mL final concentration) was added and incubated for 3 h at 37°C. Dimethyl sulfoxide was then added to dissolve insoluble formazan crystals. The absorbance was measured at 550 nm using a microplate reader (Tecan Infinite 200 Pro, Switzerland). All the experiments were repeated for three times.

### Hemolytic Assay

Red blood cells were collected by centrifugation at 1000 rpm for 10 min at 37°C. The collected red blood cells were washed multiple times with phosphate buffered saline (PBS). A 1% red blood cell solution was prepared in PBS and seeded into a 96-well microtiter plate. The red blood cells were then exposed to 100 μM bleogen pB1 or 0.1% Triton X-100 (positive control) for 1 h at 37°C. The plate was centrifuged at 1,000 rpm for 10 min at 37°C and the supernatant was transferred to a new 96-well microtiter plate. Absorbance was measured at 415 nm using a microplate reader. All the experiments were repeated for three times.

### Propidium Iodide Staining

*Candida albicans* was cultured in SOC broth before 50 μM bleogen pB1 was added and incubated for 24 h at 37°C with shaking at 350 rpm. Propidium iodide (1 μg/mL) was added and 10000 cells were evaluated using flow cytometry (BD LSRFortessa^TM^ X-20 flow cytometer, United States). All the experiments were repeated for three times.

### Statistical Analyses

Statistical comparisons were performed using GraphPad Version 6.0d (United States). Data were analyzed using one-way analysis of variance (ANOVA) followed by Newman–Keuls *post hoc* tests. Data were expressed as mean ± S.E.M and *P* < 0.05 was considered to be statistically significant.

## Author Contributions

SL, AK, and JT designed, performed, analyzed the experiments, and wrote the paper. TX performed the NMR analysis of bleogen pB1. All authors reviewed the results and approved the final version of the manuscript.

## Conflict of Interest Statement

The authors declare that the research was conducted in the absence of any commercial or financial relationships that could be construed as a potential conflict of interest.
