# Supplementary material for: Bleogens: Cactus-Derived Anti-Candida Cysteine-Rich Peptides with Three Different Precursor Arrangements
Source: Front Plant Sci. 2017 Dec 22;8:2162. doi: 10.3389/fpls.2017.02162 (PMC5743680; doi:10.3389/fpls.2017.02162)
Supplement: Supplementary file 1 [file Data_Sheet_1.PDF]

Bleogens: Cactus-derived Anti-Candida Cysteine-rich Peptides with Three Different Precursor Arrangements

Shining Loo[a], Antony Kam[a], Tianshu Xiao and James P. Tam

From the School of Biological Sciences, Nanyang Technological University, 60 Nanyang Drive, 637551, Singapore

Running title: *Cactus-derived Anti-Candida Cysteine-rich Peptides*

To whom correspondence should be addressed: Professor James P. Tam, School of Biological Sciences, Nanyang Technological University, 60 Nanyang Drive, 637551, Singapore. Email: JPTam@ntu.edu.sg

[a]These authors contributed equally to this work

Keywords: Bleogens, biosynthesis, cactus, cysteine-rich peptide, natural product, proteomics, peptides, plant

---

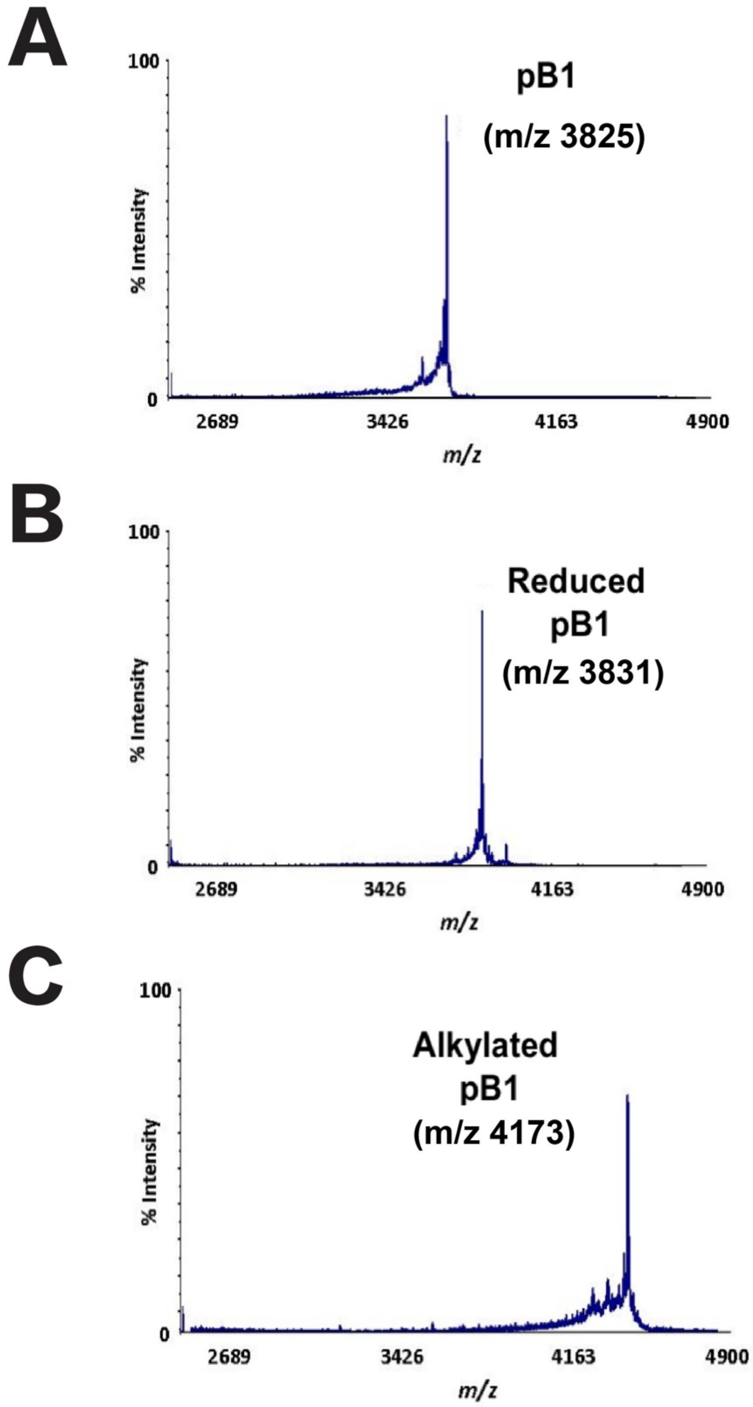

Figure S1. Reduction and alkylation of bleogen pB1

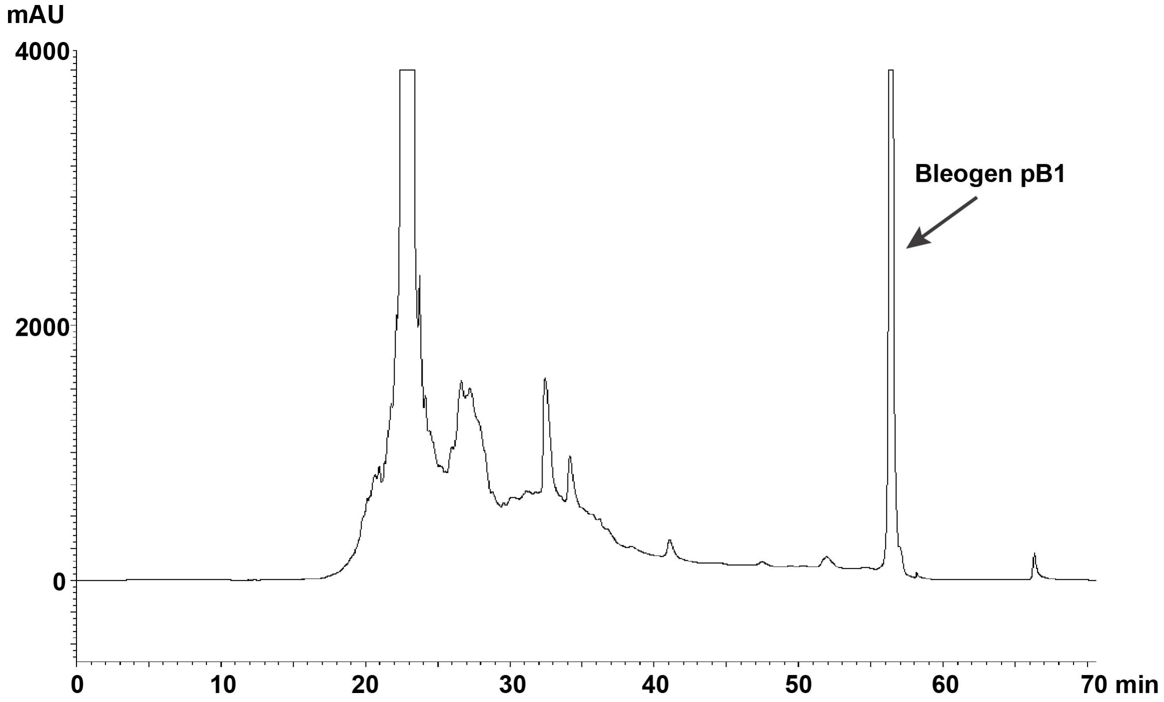

Figure S2. HPLC-DAD chromatogram of aqueous extracts of the leaves of *Pereskia bleo*.

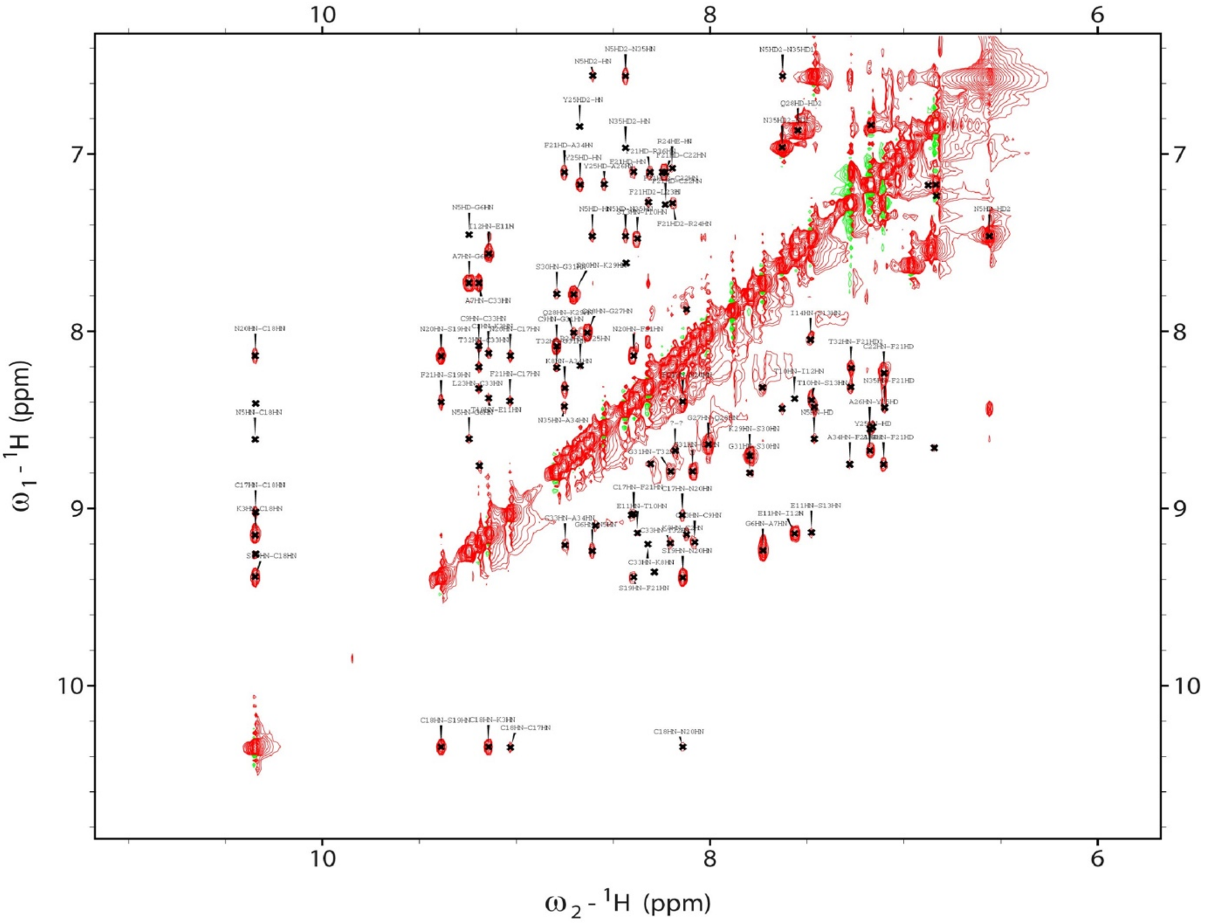

Figure S3. Chemical shift assignment of 1H, 1H-NOESY spectrum of bleogen pB1. The assignments of the NOE cross peaks between side chain protons and amide protons are displayed.

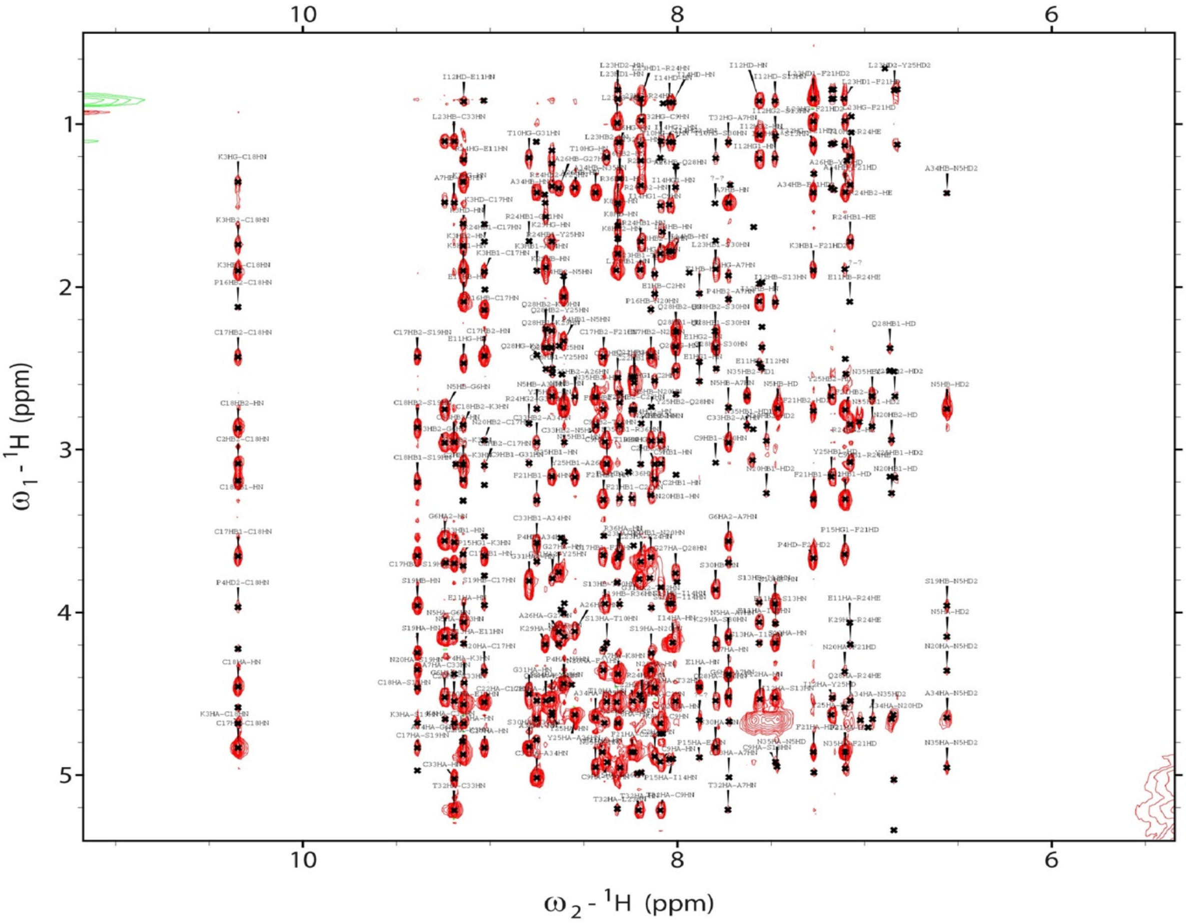

Figure S4. Chemical shift assignment of ^{1}H, ^{1}H-NOESY spectrum of bleogen pB1. The assignments of the NOE cross peaks between amide protons are displayed

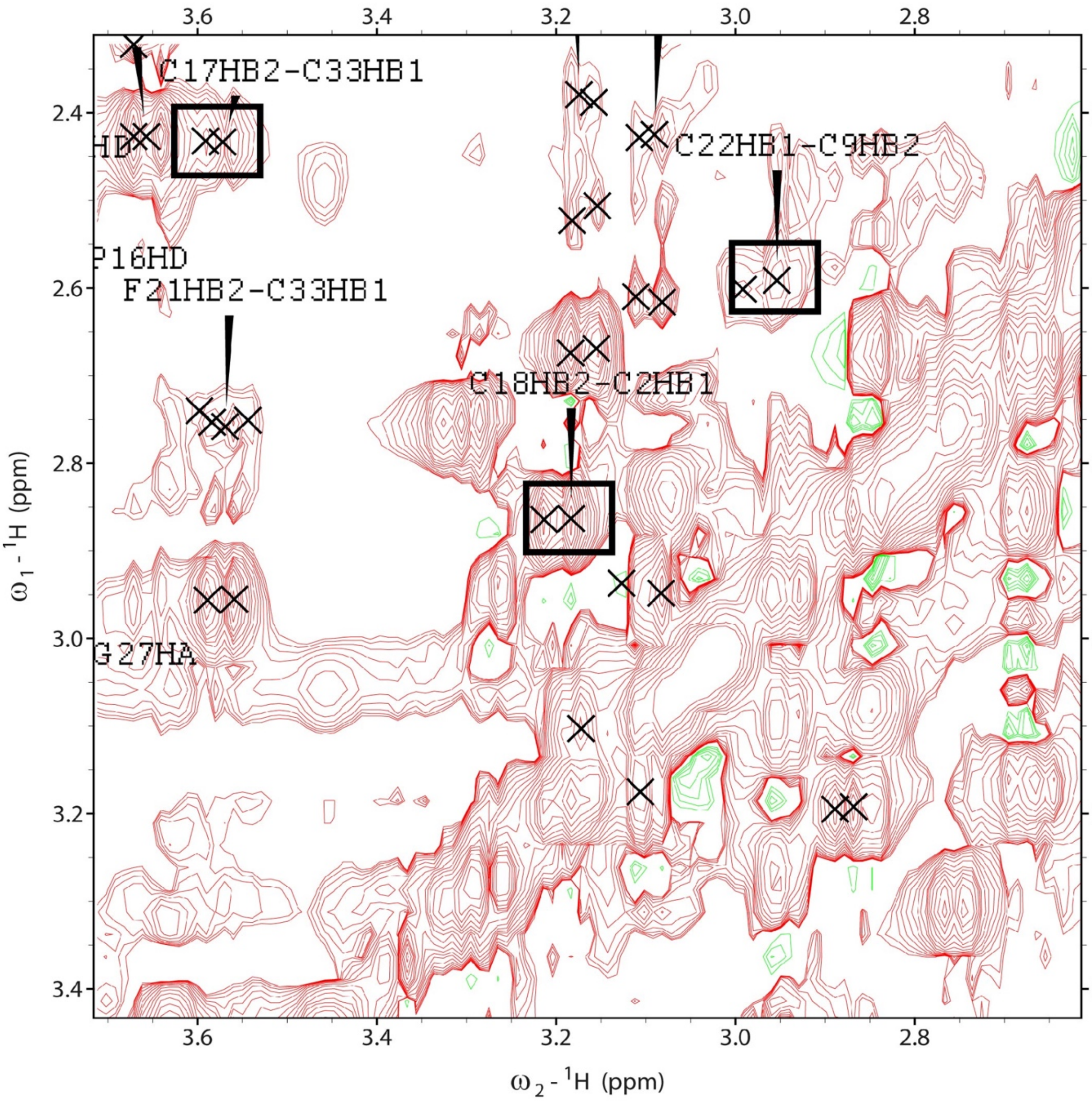

Figure S5. NOE cross peak between the Hβs of the two cysteines in each disulfide bond of bleogen pB1.

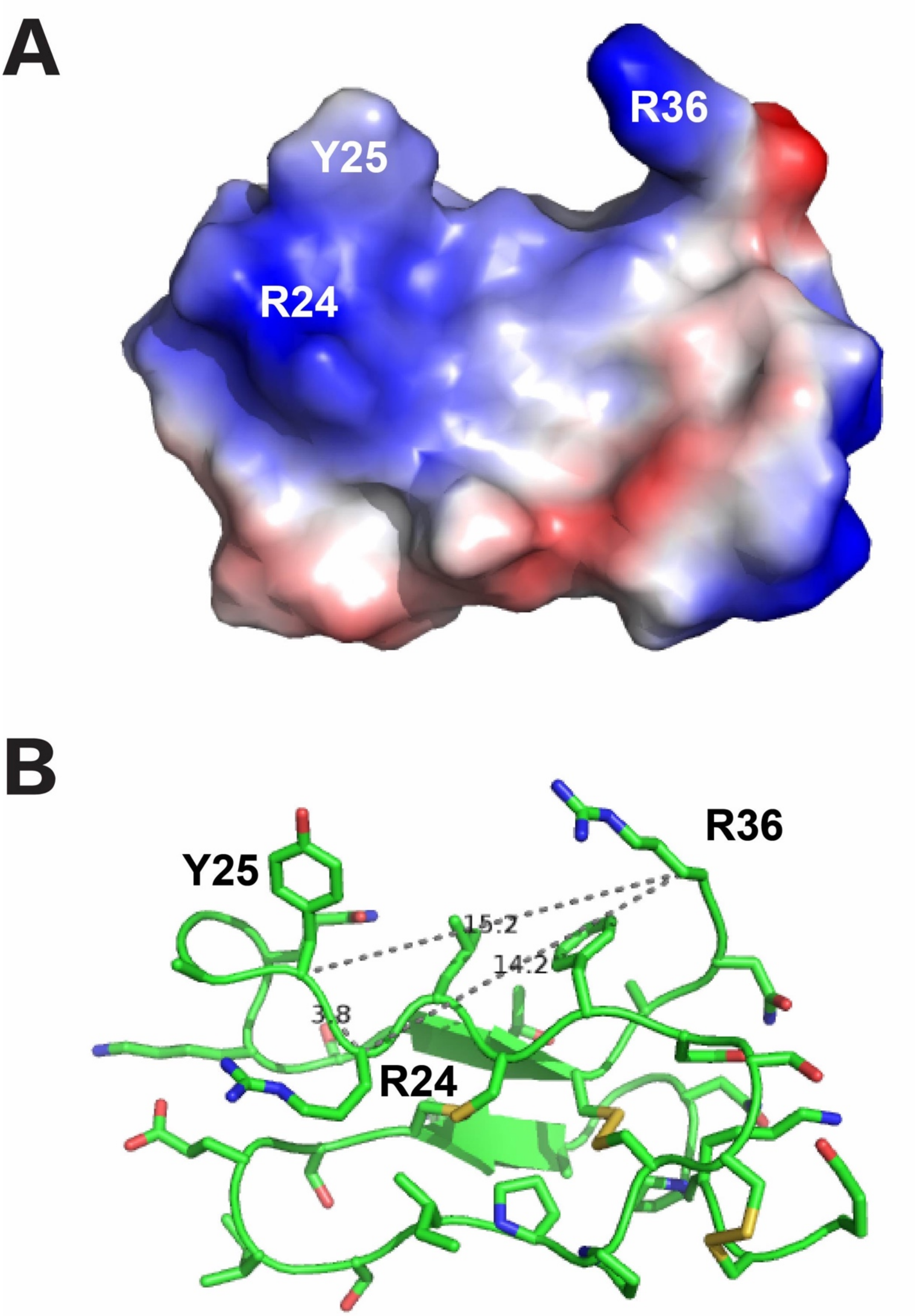

Figure S6. **(A)** Electrostatic and **(B)** cartoon view of cation-polar-cation motif of bleogen pB1.

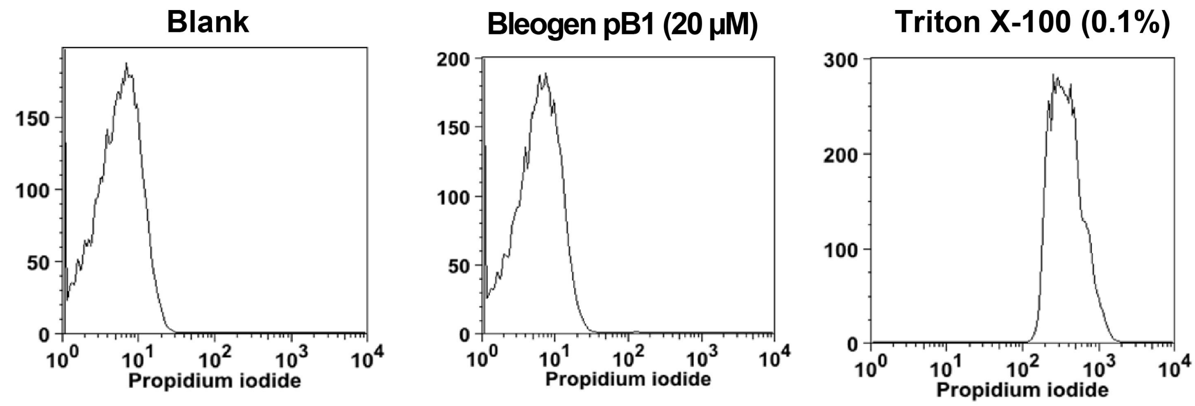

Figure S7. Propidium iodide staining of *Candida albicans* after treatment with bleogen pB1 for 20 h.

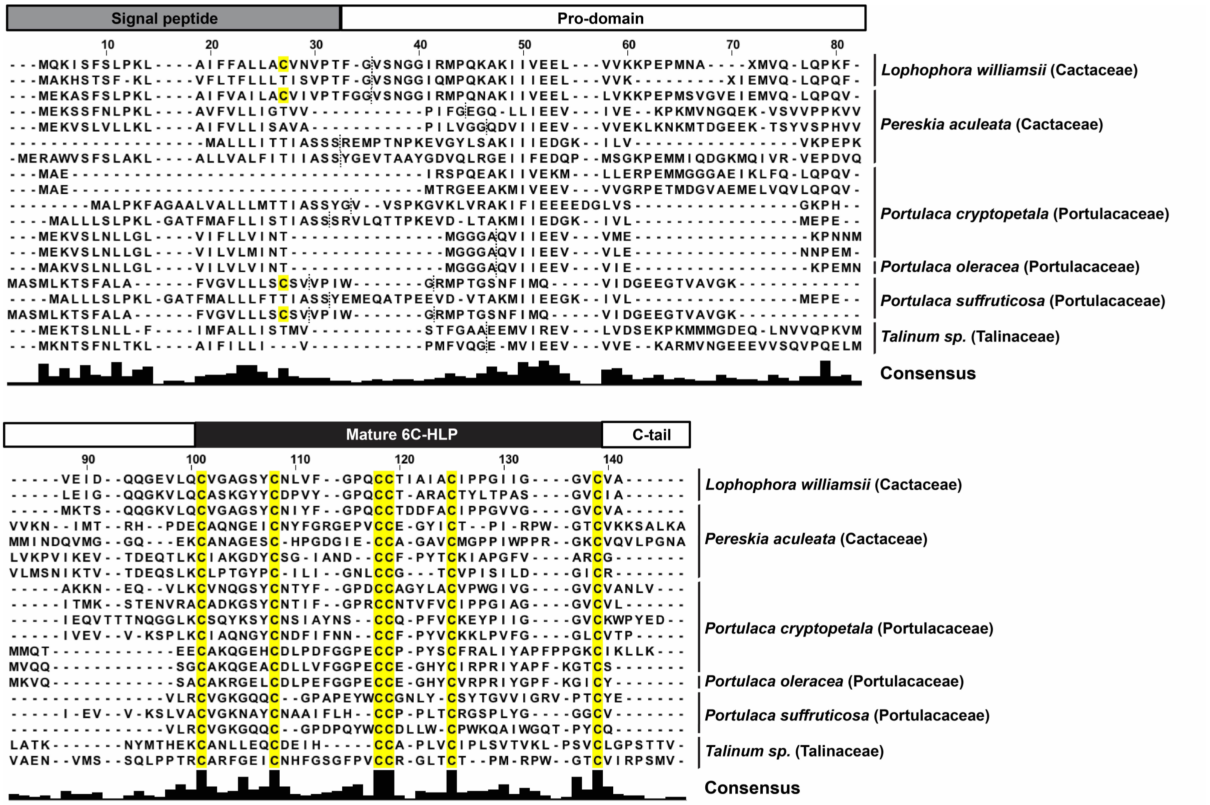

Figure S8. tBlastn search for three-domain bleogen precursor-like sequences in Onekp database. Dash lines represent signal peptide cleavage site as predicted by SignalP V4.1 and Phobius server.

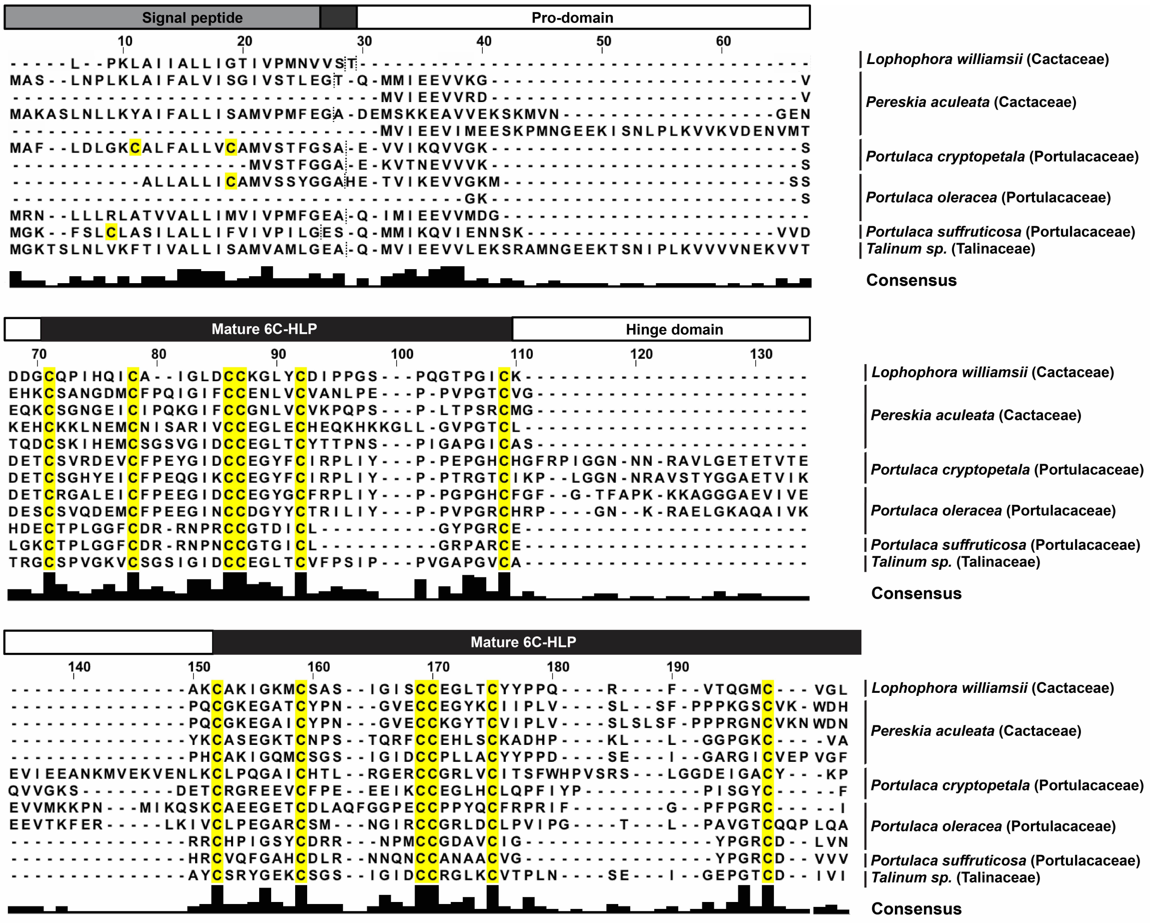

Figure S9. tBlastn search for tandemly-repeating bleogen precursor-like sequences in Onekp database. Dash lines represent signal peptide cleavage site as predicted by SignalP V4.1 and Phobius server.
